# Diagnosis and ECMO Treatment of a Critically Ill Patient With Disseminated *Mycobacterium tuberculosis*: A Case Report

**DOI:** 10.3389/fpubh.2022.938913

**Published:** 2022-07-14

**Authors:** Xiuling Shang, Hongxuan Zhang, Sheng Chen, Chen Wang, Meifu Lin, Rongguo Yu

**Affiliations:** ^1^The Third Department of Critical Care Medicine, Fujian Provincial Center for Critical Care Medicine, Fujian Provincial Key Laboratory of Critical Care Medicine, Fujian Provincial Hospital, Shengli Clinical Medical College of Fujian Medical University, Fuzhou, China; ^2^Department of Ultrasound, Shengli Clinical College of Fujian Medical University, Fujian Provincial Hospital, Fuzhou, China; ^3^Department of Pathology, Shengli Clinical College of Fujian Medical University, Fujian Provincial Hospital, Fuzhou, China; ^4^Department of Radiology, Shengli Clinical College of Fujian Medical University, Fujian Provincial Hospital, Fuzhou, China

**Keywords:** disseminated *Mycobacterium tuberculosis*, acute respiratory distress syndrome, extra-corporeal membrane oxygenation, critical care (ICU), infection

## Abstract

**Background:**

*Mycobacterium tuberculosis* infection remains a public health concern worldwide. The diagnosis and treatment of disseminated *M. tuberculosis* is very difficult, so we shared our experiences and lessons learned in this case report.

**Case Presentation:**

A 36-year-old female with a history of epilepsy presented to our hospital with fever, upper abdominal pain, muscle soreness in limbs for 7 days, and shortness of breath for 4 days. On admission, she presented with acute respiratory distress syndrome (ARDS) and liver dysfunction. Due to the critical nature of her clinical presentation, the patient was admitted directly to the Intensive Care Unit (ICU), received mechanical ventilation in prone position and VV-ECMO treatment. Her condition improved gradually, and the ECMO was removed after 7 days and she was weaned off the ventilator after 8 days. However, her fever recurred and she underwent PET-CT examination, liver contrast ultrasound, acid-fast staining and second-generation sequencing of cerebrospinal fluid, which confirmed *M. tuberculosis* infection.

**Conclusion:**

This case report briefly described the treatment and diagnosis of a critically ill patient with intra and extra-pulmonary tuberculosis infection. Timely and appropriate treatment is crucial to save lives, but the timing of ECMO treatment needs to be carefully considered for patients with ARDS caused by tuberculosis.

## Background

*Mycobacterium tuberculosis* infection remains a major public health problem and the thirteenth leading cause of death worldwide (1). The World Heath Organization (WHO) reported that 10 million people developed tuberculosis and 1.5 million people died from it in 2020 (1). *M. tuberculosis* is mainly a respiratory pathogen. However, the rate of extra-pulmonary tuberculosis infection is as high as 15%, which makes diagnosis and treatment very difficult (2). Tuberculous meningitis (TBM) is characterized by high morbidity and mortality (3). The mortality rate of confirmed tuberculosis patients requiring intensive care is as high as 68.7% (4, 5). Disproportionately higher intensive care unit (ICU) occupancy rates and lower levels of attention from medical staff make the diagnosis and treatment of severe TB patients very difficult. Previous studies reported a higher mortality rate among patients with acute respiratory distress syndrome (ARDS) caused by tuberculosis requiring mechanical ventilation in the ICU (6). Therefore, severe ARDS caused by bacterial or viral infections, trauma, etc., are very common in the ICU, but mycobacterial infection cannot be ignored.

Herein, we reported a rare case of high fever with ARDS as the initial symptoms of disseminated *M. tuberculosis* infection in a critically ill patient in the ICU of Fujian Provincial Hospital.

## Case Presentation

**Figure 1:**
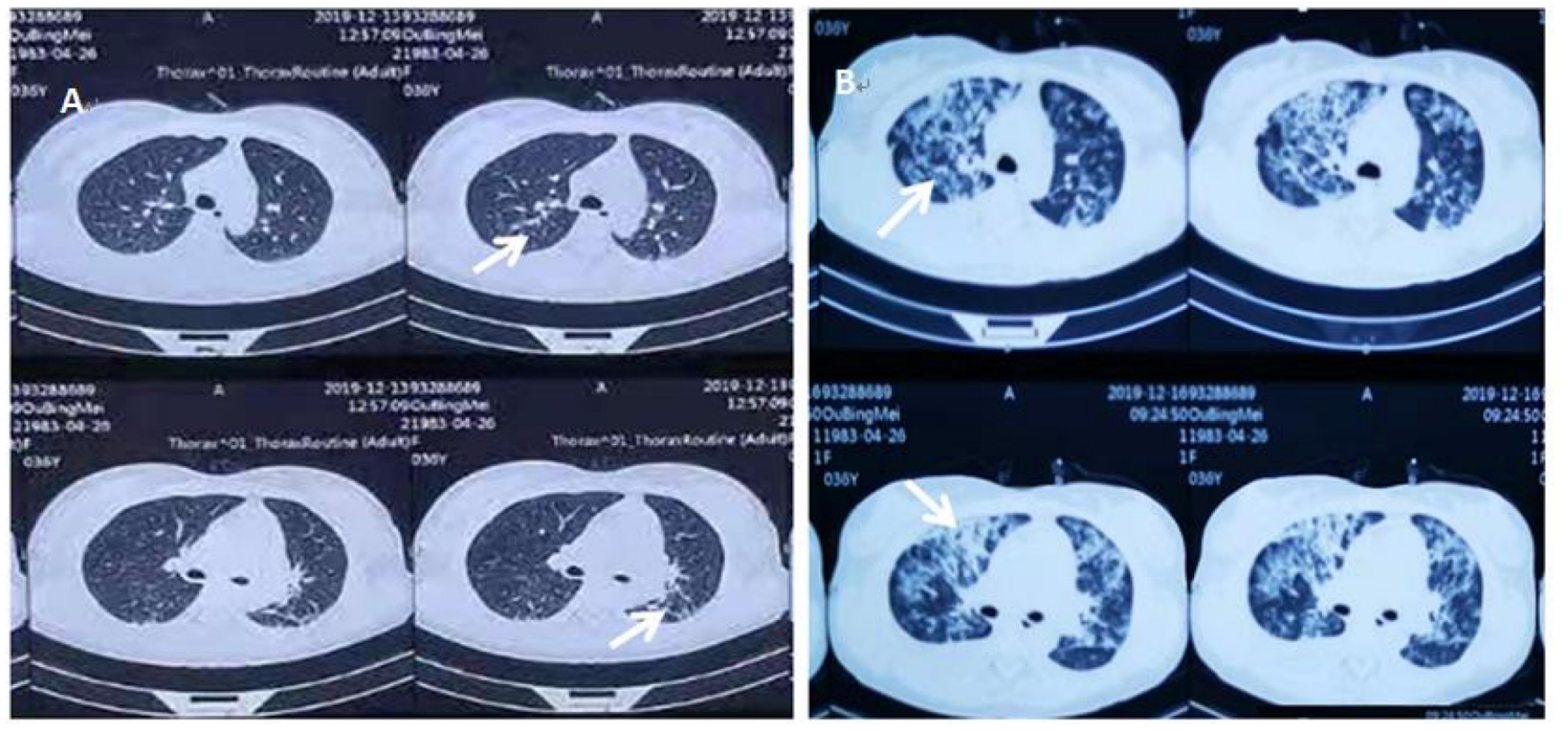
**(A)** The patient's initial chest CT examination results when she visited Zhongshan Hospital affiliated to Xiamen University on December 13, 2019, after 1 week of fever. **(B)** The repeat chest CT at Zhongshan Hospital affiliated to Xiamen University on December 16, 2019, after the patient's dyspnea worsened. White arrows indicate multiple patchy blurred shadows in bilateral lungs, which had progressed compared to initial examination.

A 36-year-old unemployed, married female, height 163 cm, weight 56 kg, of Han nationality, with a 20-year history of epilepsy presented to Fujian provincial hospital on Dec 17, 2019 with high fever for 15 days, upper abdominal pain, muscle soreness in the limbs for 7 days and shortness of breath for 4 days. Her highest armpit temperature was 41°C, accompanied by sore throat and chills. She had visited Zhongshan hospital affiliated to Xiamen university where she received oseltamivir, imipenem and cilastatin for 4 days due to leukopenia (WBC 1.44 × 10^9^/*L*) and patchy shadows on lung CT (Figure 1A), and was referred to our hospital due to progressive multiple patchy haze in bilateral lungs (Figure 1B) and dyspnea. She was taking lamotrigine (50 mg/d) and escitalopram (10 mg/d) regularly, and had no seizures in the past 3 years. She had no symptoms of dry cough or night sweats, and was otherwise healthy.

The patient had ARDS, with a respiratory rate (RR) of 36 times per min and pulse oxyhemoglobin saturation (SpO_2_) of 85% under 10 L/min oxygen supplied by an oxygen storage mask when she was referred to our emergency department. In addition, the patient had a heart rate (HR) of 110 beats per min, non-invasive blood pressure (BP) of 96/60 mmHg, and body temperature of 38.6°C. The patient was referred to the ICU, where she received sedation and invasive mechanical ventilation (VC mode, VT 360 ml, PEEP 10 cmH_2_O, FiO_2_ 100%) at 10 pm on December 7, 2019.

The patient was fully evaluated after admission to the ICU. On that night, blood gas analysis showed (FiO_2_ 100%): pH of 7.376, PaO_2_ of 74.4 mmHg, PCO_2_ of 59.4 mmHg, and oxygenation index (PaO_2_/FiO_2_) of 74.4. Blood tests showed that the white blood cell count (WBC), percentage of neutrophils (NE) and lymphocytes (L) were 3.8 × 10^9^/*L*, 45.1 and 50.7%, respectively. Procalcitonin (PCT) and C-reactive protein (CRP) were 0.25 ng/ml and 24.3 mg/L, respectively. Total bilirubin (Tbil), alanine aminotransferase (ALT) and aspartate aminotransferase (AST) were 80.97 μmol/L, 143 and 296 U/L, respectively. Yellow sclera, medium abdominal distention and liver enlargement were noted on physical examination. Therefore, femoro-femoral veno-venous extra-corporeal membrane oxygenation (V-V ECMO) and prone position were performed to improve oxygenation and lung-protective ventilation at night. The initial treatment parameters of ECMO was pump rotational speed 3,200 rpm, blood flow rate 4.0 L/min, sweep gas flow 2.5 L/min. Methylprednisolone 40 mg q8h and Meropenem 1 g q6h were empirically initiated along with negative fluid balance after taking blood and sputum samples.

**Figure 2:**
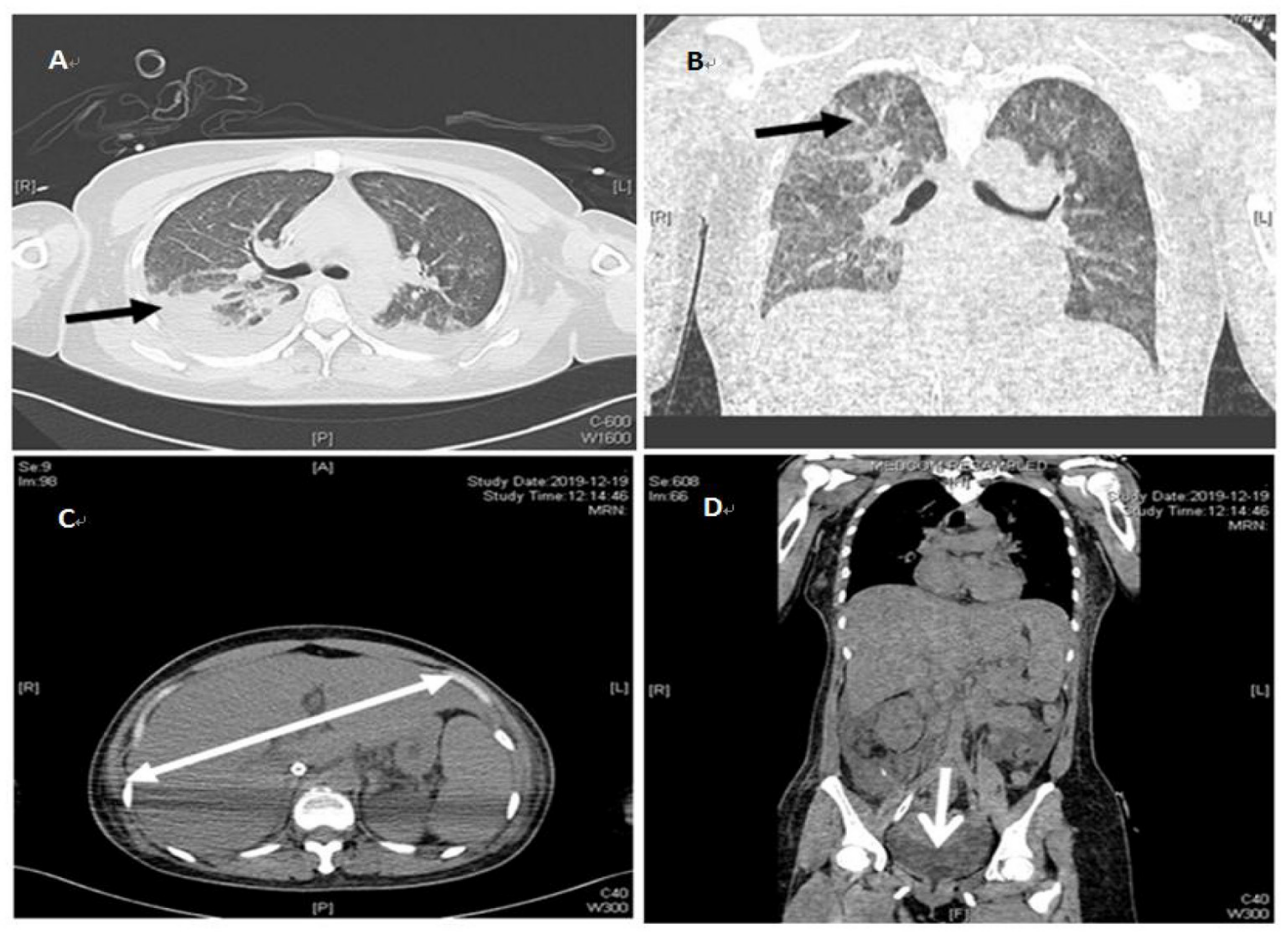
Repeat chest and abdominal CT on December 19, 2019. **(A)** indicates pleural effusion (black arrows). **(B)** indicates patchy blurring (black arrows). **(C)** indicates the enlarged liver (white k arrows). **(D)** indicates pelvic effusion (white k arrows).

**Figure 3:**
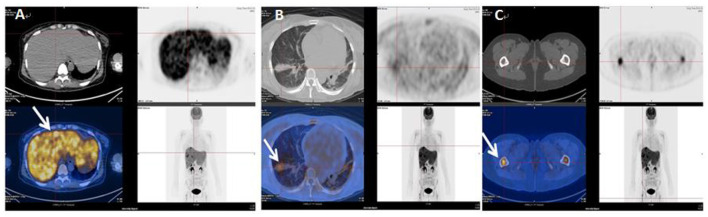
Whole body PCT-CT examination on January 8, 2020. **(A)** indicates hyper-metabolic lesions (white arrows) in the liver. **(B)** indicates hyper-metabolic lesions (white arrows) in the lungs. **(C)** indicates hyper-metabolic lesions (white arrows) in the femur.

**Figure 4:**
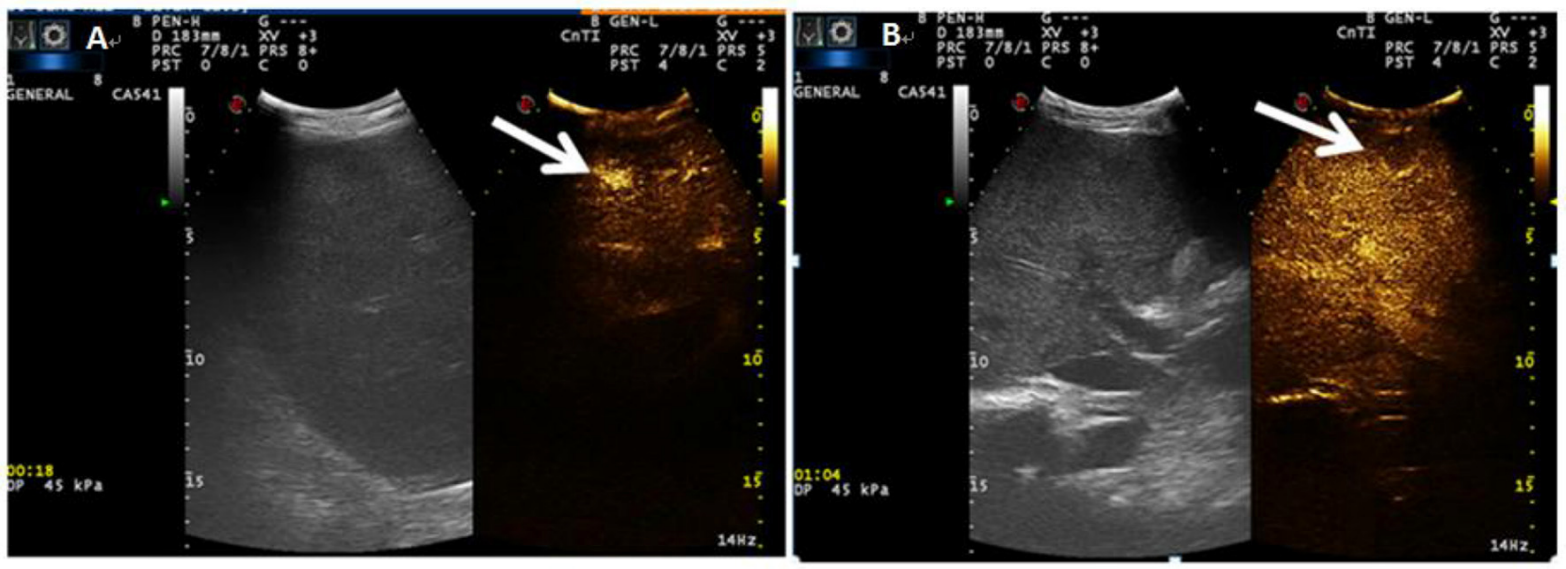
**(A)** Indicates irregular hyper-enhancement in the arterial phase of the lesion, with a transient abnormal enhancement area (white arrow) in the center. **(B)** shows the abnormal enhancement area in the delayed phase with low enhancement (white arrow) in contrast-enhanced ultrasound.

The patient's oxygenation improved and blood gas analysis showed (FiO_2_ 30%): pH of 7.47, PaO_2_ of 138 mmHg, PCO_2_ of 36 mmHg, and oxygenation index (PaO_2_/FiO_2_) of 460. Computed tomography showed multiple patchy shadows and consolidation in the dorsal region of bilateral lungs, as well as moderate pleural effusion (Figures 2A,B), enlarged liver and spleen, and moderate effusion in the abdominal cavity (Figures 2C,D). Therefore, extended prone position and physical vibration were performed on the chest to re-expand the collapsed lung. In addition, we detected numerous self-antibodies, viral antibodies and tumor markers (Table 1). On day 7, the patient's condition improved gradually and ECMO was removed, and she was weaned off the ventilator on the next day. However, fever recurred and the blood test results were as follows: 1.7 × 10^9^/*L* WBC, 60.7% NE, 32.9% L. Auto-antibodies, viral antibodies and tumor markers were negative (Table 1). So the patient underwent a whole-body PET-CT for infection screening. The results showed multiple nodular hyper-metabolic foci in the liver and lungs, slightly increased metabolism including bone marrow (Figures 3A–C). Subsequently, the patient underwent a contrast-enhanced ultrasound biopsy of the upper border of the right lobe of the liver (Figures 4A,B). Figure 4A shows irregular hyper-enhancement in the arterial phase of the lesion, with a transient abnormal enhancement area (white arrow) in the middle. Figure 4B shows the abnormal enhancement area in the delayed phase with low enhancement in contrast-enhanced ultrasound.

**Table 1:** Relevant laboratory indicators for the diagnosis of disseminated tuberculosis.

**Variable**	**Result**	**Variable**	**Result**
HIV antibodies	Negative	ANCA	Negative
Syphilis antibodies	Negative	Anti-cardiolipin antibody	Negative
Hepatitis B Antigen Test	Negative	Anti-β2 glycoprotein antibody	Negative
Hepatitis A antibodies	Negative	Antinuclear antibody	1:100 weak positive
Hepatitis E IgG	Negative	Granular positive	weak positive
Ferritin	Negative	Anti-Ro-52 antibody	weak positive
EB DNA	Negative	Anti-OJ antibody IgG	weak positive
Ceruloplasmin	Negative	Anti-Ro-52 Antibody IgG	weak positive

**Figure 5:**
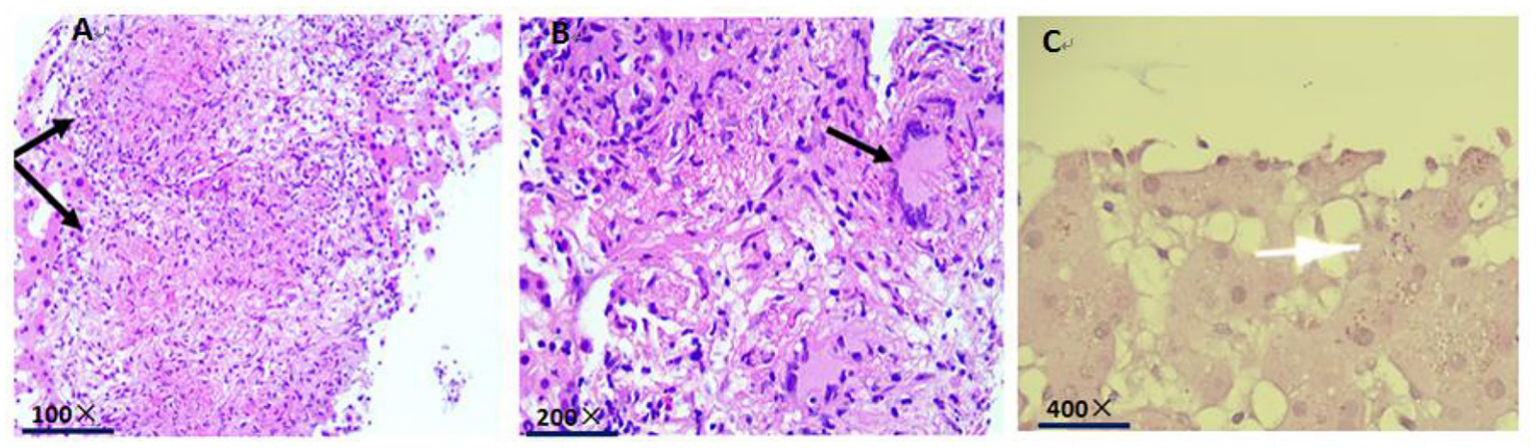
**(A)** Histopathological examination indicates fusion granulomas with caseous necrosis in the liver tissue (black arrow). **(B)** histopathological examination indicates Langhans giant cells in the liver tissue (black arrow). **(C)** acid-fast staining shows mycobacterial accumulation in the cytoplasm (white arrow).

The histopathological examination showed Langhans giant cells and fusion granulomas with caseous necrosis in the liver tissue (Figures 5A,B). Acid-fast staining revealed mycobacterial accumulation in the cytoplasm (Figure 5C). We transferred the patient to Fuzhou Pulmonary Hospital on January 19, 2020, for further diagnosis and treatment according to the requirements and regulations of the local health institution. One week later, the second-generation sequencing results of cerebrospinal fluid specimens indicated *M. tuberculosis*, the biopsy of the anterior basal segment of the right lower lobe and the posterior segment of the right upper lobe showed granulomatitis with necrosis and acid-fast staining, but PAS staining and Gram staining were negative, and the ascites and bronchoalveolar lavage fluid were also negative. Therefore, a definitive diagnosis of subacute blood disseminated pulmonary tuberculosis, tuberculous meningitis, and hepatic tuberculosis was made. The patient was treated with isoniazid 300 mg/day, ethambutol 800 mg/day, pyrazinamide 1.5 g/day and rifampicin 600 mg/day for 2 months at the Fuzhou Pulmonary Hospital, and discharged with a plan to continue long-term anti-mycobacterial tuberculosis therapy. The patient has fully recovered now.

## Discussion

Tuberculosis is the second leading infectious disease killer after COVID-19 (higher than HIV/AIDS) and the thirteenth leading cause of death worldwide (1). The incidences of tuberculosis patients admitted to the intensive care unit requiring mechanical ventilation, vasopressors, renal replacement therapy, and ECMO were 66.7, 35.9, 7.7, and 5.1%, respectively (7). VV-ECMO is an effective alternative for supportive treatment of severe ARDS, but its application in ARDS patients secondary to TB has been only noted in some case reports (8).

Timely ECMO and ventilator support facilitated the diagnosis and successful treatment of this patient. However, the diagnosis of this patient was complex, since the high fever recurred when the patient was weaned from ECMO, suggesting that the patient's etiology was not well controlled. PET-CT showed hyper-metabolic lesions in many parts of the body, such as the liver, lungs, and femur, after which contrast-enhanced ultrasound combined with puncture biopsy was performed to obtain the liver tissue with lesions. Histopathological examination of the liver revealed caseous necrosis, which confirmed the diagnosis of disseminated tuberculosis infection.

Extra-pulmonary tuberculosis of the central nervous system is a rare but significant problem due to its high disability and mortality (9). In this case, the patient developed central tuberculosis infection, without any central abnormality, which was very rare. Miliary pulmonary tuberculosis is more likely to invade the central nervous system. The detection of *M. tuberculosis* by next-generation sequencing of cerebrospinal fluid provides a new diagnostic method for central tuberculosis infection.

Tuberculosis is an important but easily overlooked management problem for patients in the ICU, and it can be cured with timely and appropriate management. Tuberculosis involving multiple organs and tissues such as the lungs, liver, bones and central nervous system is even rarer. Therefore, this case provides a diagnosis and treatment experience for a critically ill patient with intra and extra-pulmonary tuberculosis infection.

The limitation of this case report is that the mechanism of tuberculosis dissemination could not be identified although we found infections in the lungs, liver, long bones, and cerebrospinal fluid. Testing of cerebrospinal fluid and acid-fast staining of liver tissue revealed mycobacterial accumulation in the cytoplasm.

In summary, we reported a case of a severely ill patient with systemic diffuse tuberculosis infection. Although the diagnosis was finally confirmed and the treatment was successful, the process of diagnosis and treatment was complex and accompanied by a major risk of complications. Therefore, critical care physicians should consider severe ARDS caused by *M. tuberculosis* infection and monitor its spread.

## Data Availability Statement

The original contributions presented in the study are included in the article/supplementary material, further inquiries can be directed to the corresponding author.

## Ethics Statement

The studies involving human participants were reviewed and approved by K2020-05-037. The patients/participants provided their written informed consent to participate in this study.

## Author Contributions

HZ collected the data. XS drafted the manuscript. RY revised the manuscript. All authors read and approved the final version of the manuscript.

## Conflict of Interest

The authors declare that the research was conducted in the absence of any commercial or financial relationships that could be construed as a potential conflict of interest.

## Publisher's Note

All claims expressed in this article are solely those of the authors and do not necessarily represent those of their affiliated organizations, or those of the publisher, the editors and the reviewers. Any product that may be evaluated in this article, or claim that may be made by its manufacturer, is not guaranteed or endorsed by the publisher.

## Abbreviations

ICU: Intensive Care Unit
ECMO: Extra-Corporeal Membrane Oxygenation
ARDS: Acute Respiratory Distress Syndrome
AIDS: Acquired Immune Deficiency Syndrome.
